# Direct Reprograming to Regenerate Myocardium and Repair Its Pacemaker and Conduction System

**DOI:** 10.3390/medicines5020048

**Published:** 2018-06-04

**Authors:** Saritha Adepu, Erik F. J. Oosterwerff, Vincent M. Christoffels, Gerard J. J. Boink

**Affiliations:** 1 Department of Medical Biology, Academic Medical Center, University of Amsterdam, 1105 AZ Amsterdam, The Netherlands; 2 Department of Cardiology, Isala Klinieken, 8025 AB Zwolle, The Netherlands; 3 Department of Cardiology, Academic Medical Center, University of Amsterdam, 1105 AZ Amsterdam, The Netherlands

**Keywords:** direct reprogramming, cardiac conduction system, biological pacemaker

## Abstract

The regenerative medicine field has been revolutionized by the direct conversion of one cell type to another by ectopic expression of lineage-specific transcription factors. The direct reprogramming of fibroblasts to induced cardiac myocytes (iCMs) by core cardiac transcription factors (Gata4, Mef2c, Tbx5) both in vitro and in vivo has paved the way in cardiac regeneration and repair. Several independent research groups have successfully reported the direct reprogramming of fibroblasts in injured myocardium to cardiac myocytes employing a variety of approaches that rely on transcription factors, small molecules, and micro RNAs (miRNAs). Recently, this technology has been considered for local repair of the pacemaker and the cardiac conduction system. To address this, we will first discuss the direct reprograming advancements in the setting of working myocardium regeneration, and then elaborate on how this technology can be applied to repair the cardiac pacemaker and the conduction system.

## 1. Introduction

The regenerative medicine field has been revolutionized by the direct conversion of one cell type to another by exogenous of over expression of lineage-specific transcription factors (TFs). The direct reprogramming field finds it origin in the ground-breaking work by Yamanaka and colleagues who demonstrated that pluripotent stem cells (PSCs) can be generated by forced overexpression of a combination of transcription factors (TFs; typically, Oct4, Sox2, cMyc and Klf4) in somatic cells, such as fibroblasts [1]. After the initial development of these induced PSCs (iPSCs), a large number of research groups around the world have successfully implemented the technology in their laboratories, underlining the robustness of this approach. iPSCs have successfully been used for the generation of a large variety of cell types, including cardiomyocytes (CMs). More recently, efficient protocols have also been designed to direct iPSCs to specific cardiomyocyte subtypes, including atrial, ventricular and nodal-like cells [2,3,4]. However, for therapeutic applications, transplantation of iPSC-derived cells remains challenging as a consequence of functional immaturity, potential tumorigenicity, and additional issues related to the transplantation efficiency and risk of immune-rejection [5]. To overcome the challenges of cell-based therapies, the regenerative field has been expanded by exploration of the direct in vivo conversion of one cell type into another. This direct reprogramming was first demonstrated in mouse pancreas by direct conversion of α-cells into insulin producing β-cells [6]. Thereafter, direct reprogramming of fibroblasts into various target cells has been reported, including neurons, CMs, and hepatocytes [7,8,9]. Several independent research groups have successfully demonstrated direct reprogramming of fibroblasts towards CMs, employing a variety of approaches that rely on TFs, small molecules, and micro RNAs (miRNAs). More recently, this technology is being considered for local repair of the pacemaker and the cardiac conduction system (CCS).

Cardiac pacemaker activity is primarily generated by the sinoatrial node (SAN) [10,11]. There is substantial understanding of the complex interactions of the different ion channels and intracellular proteins that underlie pacemaker function. Two key discoveries have been the funny current (‘*I*_f_’) and the associated so called ‘Membrane-clock’, and the ‘Ca^2+^-clock’ mechanism that was later integrated with the former in the coupled-clock concept [12]. The depolarization initiated in the SAN subsequently activates the atria, traverses the atrioventricular node (AVN), AV bundle (AVB; also referred to as bundle of His), and bundle branches, and subsequently activates ventricular working myocardium via the ventricular Purkinje network. This triggers a coordinated contraction of the atria and ventricles by propagation of electrical impulses via the CCS, while maintaining an autonomically-controlled heart rhythm. Any prolonged/persistent disturbance in this impulse generation and transmission can result in CCS dysfunction.

The major pacemaker and CCS disorders are Sick Sinus Syndrome (SSS) and complete heart block. SSS primarily results from abnormalities in impulse formation within the SAN and propagation at the interface between SAN and the right atrium [13]. Complete heart block can occur at the level of the AVN, or just below this node within the proximal AVB [14]. Common causes for SAN or AVN dysfunction include aging, ischemic heart disease and various underlying cardiac pathologies. These factors can cause ion channel remodeling [15] and increase fibrosis, resulting in nodal dysfunction and disease-causing bradycardia (slow heart rates) [16].

The current standard of therapy for bradycardias includes the implantation of electronic pacemakers. Some of the shortcomings of these devices are inadequate sensitivity to autonomic modulation, limited battery life, and inappropriateness for many pediatric patients and patients with congenital heart diseases. These disadvantages have fostered the development of biological approaches to repair disorders of the cardiac pacemaker and CCS. In order to develop novel therapies that can bypass these shortcomings, several gene and cell therapy approaches have been explored in short-term animal models of bradycardia [17]. Novel regenerative therapies may be developed by using direct reprogramming, aiming to target cardiac myocytes as a source for pacemaker cell regeneration or to convert excessive fibrosis into pacemaker and CCS-like cells. To address this, we will first discuss the direct reprograming advancements in settings of working myocardium regeneration, and next elaborate on how this technology may be applied to repair of the cardiac pacemaker and CCS.

## 2. Direct Reprogramming for Cardiac Regeneration

Ieda et al. first reported the direct in vitro conversion of neonatal mouse cardiac fibroblasts (MCFs) and tail tip fibroblasts (TTFs) into induced cardiomyocyte-like cells (iCMs) in vitro by overexpressing a combination of three core cardiac TFs (Gata4, Mef2c, Tbx5), together called GMT. To identify these TFs, they used fibroblasts isolated from an α-MHC-GFP reporter mouse line that marks cardiomyocytes. These fibroblasts lack GFP expression upon isolation but start to express GFP once they have successfully been reprogrammed towards the cardiac lineage. To identify the minimally required combination of most potent factors for directing fibroblasts towards CMs, they employed a subtraction strategy beginning with a group of 14 TFs that were selected based on their importance during cardiac development. Next, the most potent TF combination was identified by serially removing individual TFs from the initial cocktail of 14-factors (comparable to the method originally used by Yamanaka et al., Cell, 2006 [1]), identifying GMT as the most promising combination. Flow cytometry was used to quantify GFP expressing iCMs at week 1, 2 and 4 as a measure of reprogramming efficiency. The percentage of cTnT expressing cells in GFP^+^ iCMs indicated that the efficiency of iCM generation was low (~5% in MCFs and ~2.5% in TTFs), emphasizing that only small subsets of α-MHC expressing GFP^+^ cells have differentiated towards *bonafide* cardiomyocytes. Moreover, even a smaller fraction of iCMs (0.1%) exhibited spontaneous contractions after 4 to 5 weeks in culture. The GMT cocktail also reprogrammed TTFs (albeit with lesser efficiency), indicating that non-cardiac fibroblasts also transdifferentiate to iCMs, eliminating the possibility of cardiac progenitor cells being the sole source of effective reprogramming towards iCMs. Overall, this study provided important proof-of-concept for the transdifferentiation of fibroblasts to iCMs, but the in vitro process is still relatively slow and inefficient [9].

The hypothesis that direct reprogramming may be more effective in vivo, has led to the investigation of several related strategies. The first study that confirmed this hypothesis came from overexpressing the cardiac TFs Gata4, Nkx2-5 and Tbx5 together with the chromatin remodeling protein Baf60c in mouse embryos. This combination of factors was shown to reprogram non-cardiac mesoderm towards beating CMs, while also indicating redundancy for Nkx2-5 in this setting [18]. Interestingly, this combination of factors had failed to produce effective reprogramming in vitro [9], suggesting the importance of the in vivo microenvironment.

To investigate direct reprogramming in the heart it was important to identify a reliable vector system that would allow for specific transduction of CFs in vivo. For this purpose, the retroviral vector system appeared reasonably suitable, as this would preferentially transduce dividing cells limiting its efficacy in the heart to non-CMs. Qian and colleagues were among the first to use this system in their approach to overexpress GMT in a mouse model of myocardial infarction (MI), induced by coronary artery ligation. Lineage tracing experiments in reporter mice using Cre-recombinase driven by a periostin-locus (specific for fibroblasts) showed that transdifferentiated iCMs were indeed obtained from fibroblast origin. qPCR analysis of iCMs for cardiac enriched genes revealed their similarity to mature CMs. The in vivo reprogramming efficiency of GMT was shown to be similar to the in vitro situation (10–15% based on α-MHC-GFP^+^ expressing cells) but the iCMs more closely resembled a mature phenotype. Functional studies indicated a moderate improvement in cardiac function (Table 1) and reduction in infarct size, when compared to dsRed injected control group 12 weeks post MI [19]. These outcomes were further confirmed in a follow-up study that investigated GMT overexpression using a single polycistronic vector (Table 1) [20].

Because cardiac regeneration is one of the ultimate goals of in vivo direct reprogramming, it was logical to also optimize the approach in adult cells. The Olson group, therefore, screened additional TF combinations in adult MCFs obtained from α-MHC-GFP reporter mice. This screen employed six core cardiac TFs that play important roles during heart development. Using this method, they found the combination of GMT together with Hand2 (GMTH) to be most effective. The initial screen performed by Ieda et al. also indicated a contribution of Hand2 in the generation of GFP^+^ iCMs. However, its removal did not decrease the percentage of iCMs and hence it was excluded in their further screens. FACS analysis for α-MHC-GFP^+^/cTnT^+^ showed positive iCMs in 6.8% of CFs with GMTH as compared to 1.4% of CFs with GMT. Motivated by these results, the GMTH cocktail was subsequently tested in vivo in α-MHC-GFP transgenic mice subjected to MI. Here outcomes suggest possible superiority for GMTH as compared to GMT (Table 1) [21]. Although the GMT/GMTH TF-based methods effectively silenced the fibroblast phenotype, and switched on a global cardiac gene program, the overall reprogramming efficiency was low, providing a major challenge for further translation. Moreover, the iCMs generated by direct reprogramming appear still immature in terms of their electrophysiological profile [22]. As a result, extensive research efforts have now been focused on further optimizing the reprogramming efficiency and maturation of the obtained iCMs.

### 2.1. Further Optimizing the TF Cocktail

Similar to Ieda et al., S. Protze et al. screened for triplet combinations of 10 candidate factors in comparison with GMT to generate iCMs from Myh6-tdTomato reporter mice using mouse embryonic fibroblasts (MEFs) and neonatal CFs. The read-out for reprogramming efficiency was activation of a broader range of cardiac-specific genes. This screen yielded Tbx5, Mef2c and Myocardin (TMM) as the most potent combination for iCM generation with a higher activation of multiple cardiac genes (2.5% vs. 2.2%, for TMM vs. GMT). However, this combination did not result in the generation of beating CMs [23]. Another study employed a GCaMP calcium reporter lentiviral vector to quantify reprogramming efficiency in vitro. Via this vector, GCaMP activity is driven by cardiac Troponin T promoter, thereby enabling monitoring of calcium flux in iCMs in vitro. Unlike flow cytometry, immuno-histochemistry or qPCR-based cardiac gene expression analysis, this approach relied on functional calcium activity of successfully reprogrammed cells as the primary read-out. Gata4, Mef2c, Tbx5, Hand2, Nkx2.5, (GMTHN), along with GMT and a variety of other published TF cocktails were overexpressed in MEFs and adult CFs via lentiviral gene transfer. In this setting, the GMTHN combination appeared to be >50 fold more efficient than the GMT cocktail in terms of the cardiac transcriptome and number of spontaneously contracting cells. This study also demonstrated that long-term overexpression of reprogramming factors is not a prerequisite to maintaining the cardiac phenotype of reprogrammed cells. This was demonstrated by using doxycycline (Dox)-inducible vectors that can be employed to inactivate transgene expression by removing Dox from the culture medium [24].

The Olson group additionally screened for protein kinases that can boost cardiac reprogramming by altering the relevant intracellular signaling pathways. Their protein kinase library results showed that TTF reprogramming was accelerated by addition of Akt1 to GMTH (GMTHA). The mechanism of action is not entirely understood but Akt1 seems to enhance fibroblast reprogramming through pathways involving IGF1, P13K, mTORC1 and Fox3a signaling [25]. The same group recently reported further enhancement of reprogramming efficiency by addition of the TF ZNF281 to the GMTHA cocktail. Co-CHIP- and RNA-sequencing analysis revealed that ZNF281 enhanced cardiac reprogramming by associating with Gata4 on cardiac enhancers, and by inhibition of inflammatory pathways [26].

### 2.2. Use of Small Molecules to Improve Reprogramming

In the context of reprogramming, small molecules are typically defined as chemical compounds that have the potential to alter cellular signaling pathways. This can be achieved by their exogenous in vitro or in vivo application (for a certain duration of time) to cells that are in the process of reprogramming. Advantages of the use of small molecules include their ease of application and strict temporal control, which make them highly effective in optimizing the process of TF-mediated reprogramming. Moreover, there is emerging knowledge on guiding the differentiation processes of PSCs and cardiac progenitor cell-derived cells. This has illustrated the usefulness of small molecules in modulating signaling pathways that are important for cardiomyocyte development and maturation [27,28,29]. Hence, the use of small molecules was a logical next step to improve direct reprogramming. The TGFβ signaling pathway is of crucial importance for maintenance of the fibroblast phenotype, so its inhibition has provided an attractive target in direct reprogramming. The addition of the TGFβ inhibitor SB431542 to a TF cocktail (Hand2, Nkx2.5, Gata4, Mef2c and Tbx5) indeed increased the reprogramming efficiency from 5% to 16% (as measured by spontaneous calcium oscillations; [30]). Similarly, inhibition of profibrotic signaling pathways involving TGFβ signaling or Rho-kinase associated pathways (using the small molecules A83-01 and Y-27632 respectively) also appeared useful in improving direct reprogramming based on GMTH [31].

In an effort to further identify highly effective small molecules and/or potential novel pathways Mohamed et al. employed a high throughput screen using a library of 5500 chemical compounds. This screen was ultimately narrowed down to two potent small molecules, the previously identified TGFβ inhibitor SB431542 and the Wnt signaling inhibitor XAV939. GMT overexpression combined with a cocktail of these two inhibitors (GMTc) accelerated in vitro reprogramming as compared to GMT alone. Beating cells were now observed as early as eight days post transduction (as compared to 6–8 weeks with GMT) and the reprogramming efficiency was increased by 8-fold. In vivo administration of GMTc in a mouse model of MI increased the ejection fraction (EF) one week after its application, as compared to GMT alone (EF GMTc ~45% vs. EF GMT ~35%) and persisted throughout the 12-week study (EF GMTc ~40% vs. EF GMT ~35%). Although this screen failed to identify novel pathways, it provided a platform for further high-throughput testing and emphasized the robustness of the TGFβ and Wnt signaling pathways [32].

### 2.3. Epigenetic Factors and RNA Splicing in iCM Generation

An important prerequisite for effective reprogramming is the need to overcome epigenetic barriers. As a result, research has been focused on finding epigenetic factors/modifiers that can improve direct reprogramming. Liu et al. characterized the differential expression of histone marks and DNA methylation in mouse fibroblast transduced with a polycistronic MGT vector. Their results show an increase in transcriptionally active trimethylated histone H3 of lysine 4 (H3K4me3) and a decrease in transcriptionally inactive trimethylated histone H3 of lysine 27 (H3K27me3) at cardiac promoters, seen already three days after the initiation of reprogramming. In parallel, reduced H3K4me3 and increased H3k27me3 was observed at fibroblast promoters. The DNA methylation status of cardiac gene promoters (*Myh6* and *Nppa*) revealed demethylation of CpGs. These results clearly indicate enrichment in cardiac genes and suppression of fibroblast specific genes [33]. In another study Zhao et al. screened for epigenetic factors that could potentially regulate iCM reprogramming. Their screen identified Bmi1 (a key component of polycomb repressive complex gene, PRC1) as a critical inhibitor in iCM generation. Bmi1 regulates cell senescence and proliferation via p16Ink4a, P19Arf, and P53 loci in MEFs and stem cells [34,35]. However, in iCM generation, Bmi1 appears to represses cardiogenic gene activation by direct binding to regulatory elements. shRNA mediated knockdown of Bmi1 resulted in induction of H3k4me3, thus enhancing iCM generation, sarcomere formation and spontaneously beating cells in neonatal and adult fibroblasts. Bmi1 suppression also induced Gata4, thereby removing the need for exogenous Gata4 expression to induce reprogramming [36]. Another study screened for 47 cardiac specific epigenetic factors and TFs in MEFs [37]. This screen identified H3K4methyltransferase Mll1 and related factor Men1 as inhibitors of iCM conversion. Inhibition of these two factors by small molecules (MM408 and MI503) resulted in enhanced iCM generation. Mll1 acts via its target gene Ebf1 driving adipocyte differentiation and its inhibition by MM408 resulted in suppression of adipocyte differentiation of fibroblasts. This study suggests the importance of suppression of other non-myocyte lineage conversion of fibroblasts during iCM induction [38].

The regulation of RNA splicing may be of equal importance in somatic cell reprogramming. RNA splicing is defined as post-translational modification of nascent pre-mRNA to form mature mRNA. Changes in global splicing factors have been noted in direct conversion of fibroblasts to iCMs [39]. Qian et al. performed single-cell RNA sequencing of day 3 reprogramming mouse fibroblasts to study early global transcriptome changes. This analysis revealed down regulation of several alternative-splicing factors at this stage. Among them, Ptbp1 has been identified as a critical barrier in attaining iCM phenotype and its knock down by shRNA resulted in enhancement of iCM generation. This study underlines the importance of RNA splicing mechanisms in regulating cell differentiation during reprogramming [40]. In order to identify the epigenetic and splicing factors involved in iCM generation, the same group performed shRNA-mediated loss of function assays that identified splicing factors Sf3a1 and Sf3b1 to be essential for iCM conversion. In contrast, knock down of Zrsr2, Bcor and Stag2 resulted in enhanced iCM generation [41].

### 2.4. Direct Reprogramming of Human Fibroblasts

The ultimate goal of direct reprogramming is the treatment of human subjects. The above-mentioned TFs and small molecules partially transdifferentiated mouse fibroblasts, but in general failed to effectively reprogram human CFs (HCFs). This suggests that human fibroblasts are more resistant to reprogramming and/or require additional optimization of the minimal required factors. Micro RNAs are small, noncoding RNAs that can fine-tune a broad range of cellular processes and also importantly regulate cellular development and differentiation. For example, they are important for post-transcriptional regulation of gene expression in stem cells [42,43]. This suggested their potential usefulness to further optimize transdifferentiation of human fibroblasts towards a cardiac fate. Based on this notion, Jayawardena et al. screened several miRNAs that were important in cardiac muscle development and differentiation. They observed that forced expression of microRNAs MiR-1, -133, -208 and -499 could reprogram mouse fibroblasts both in vitro and in vivo. Overexpression of these miRNAs by transient transfection of MEFs, neonatal CFs and adult CFs resulted in iCMs that exhibited cardiac gene expression, sarcomere organization, calcium oscillations, and mechanical contractions in vitro [44]. These findings demonstrate that miRNAs can indeed promote direct reprogramming of mouse fibroblasts. To test if miRNAs can also enhance human fibroblast reprogramming, miR-1 and -133 were overexpressed in combination with Gata4, Hand2, Tbx5 and myocardin. This induced cTnT expression in approximately 13% of cells and occasionally spontaneously beating cells after culturing them for 11 weeks (Table 2). It appears that miR-1 promotes the sarcomere development while miR-133 suppresses the smooth muscle gene program thus enhancing reprogramming efficiency [45].

Another study investigated effective combinations of TFs to transdifferentiate HCFs and dermal fibroblasts (DFs) into iCMs. The reprogramming efficiency was assessed by the ability to activate a wider range of cardiac enriched genes and also cause spontaneous Ca^2+^ oscillations, the functional measure for iCMs generated in vitro. The addition of Mesp1 and myocardin to the GMT cocktail induced a cardiac phenotype in HCFs and DFs. The reprogrammed cells expressed multiple cardiac genes, and exhibited sarcomeric organization and spontaneous calcium oscillations. Approximately 6% of iCMs showed α-actinin expression (Table 2) [46]. Addition of miR-133 to the GMT or GMT+ Mesp1 TF cocktails promoted transdifferentiation of mouse and human fibroblasts. Here enhancement in reprogramming efficiency was shown to involve repression of Snail1 signaling (which regulates epithelial to mesenchymal transition) and inhibition of the fibroblast phenotype [47].

The group of Singh and colleagues showed that addition of Hand2 and myocardin or MiR-590 to the GMT cocktail reprogrammed porcine and human fibroblasts into iCMs. Approximately 5% of the iCMs exhibited cTnT expression together with MYH6 and TNNT2 upregulation (Table 2). The mechanism by which miR-590 enhances cardiac reprogramming is by suppressing Sp1, which is known for its inhibition of cardiac-specific genes. This study suggests that miR-590 can act as a substitute for Hand2 and myocardin, thereby eliminating the need for additional TFs other than GMT to reprogram human or porcine fibroblasts. Interestingly, addition of Hand2 and myocardin or miR-590, to GMT was equally effective in reprogramming both porcine and human fibroblasts, thus highlighting the translational importance of porcine (or comparable large animal) studies in preparation for eventual human trials [48].

Finally, Fu et al. used human H9ESC derived fibroblasts (containing a α-MHC-driven mCherry reporter), neonatal and skin fibroblasts for additional iCM optimization studies. They reported effective reprogramming for a five-factor combination containing GMT, ESRRG and MESP-1. Interestingly, addition of MYOCD and ZFMP2 further enhanced reprogramming efficiency. This seven-factor combination resulted in cTNT expression in approximately 5% of iCMs. Global gene expression data of cTNT+ iCMs showed a shift from fibroblasts to CM profile [50]. The same group recently showed that addition of TGFβ and Wnt inhibitors to the 7F combination enhanced the iCM conversion of an adult HCF cell line from 5% to 12% in conjunction with enhanced sarcomere formation [32]. All together, these studies illustrate the potential of jointly using a TFs, miRs and small molecules in an effort to improve human iCM generation.

## 3. Reprogramming to Regenerate the CCS

The progress made by direct reprogramming of fibroblasts using a combination of core cardiac TFs, small molecules, and miRNAs encourages the use of this technology to regenerate the pacemaker and the CCS. Previous findings indicate that GMT-based reprogramming generates mostly ventricular-like CMs [9], while GMT/miR-133 generates mostly atrial-like CMs [47] and GHMT-based reprogramming generates a mixed population of induced atrial myocytes (iAMs), induced ventricular myocytes (iVMs) and induced pacemaker cells (iPMs; ~33% each; [51]) illustrating the possibility of generating specific CM subtypes using direct reprogramming.

The TFs Shox2, Isl1, Tbx3, and Tbx18 play a crucial role in SAN formation during embryonic development [52]. A prerequisite for CCS repair by direct reprogramming is the identification of key factors required for the generation of pure populations of relevant CCS type cells. A potential starting point towards achieving this goal would be overexpression of one or more critical TFs in cells that are closely related. Tbx3 is exclusively expressed in the CCS, and, during development acts as a strong inducer of the pacemaker gene program while repressing chamber-specific genes [53]. Therefore, our initial approach was to overexpress Tbx3 in working myocardium. When Tbx3 was overexpressed in atrial myocytes during embryonic development, ectopic pacemaker activity could be induced in approximately 50% of the atria isolated from transgenic mice with constitutive Tbx3 overexpression. Moreover, patch-clamp studies on single myocytes isolated from these atria demonstrated spontaneous activity in about 50% of the cells that displayed hallmark features of SAN cells, such as a slow upstroke velocity and phase 4 diastolic depolarization [54]. In a subsequent study, we investigated if Tbx3 could also convert terminally differentiated CMs towards the pacemaker lineages [55]. This was studied using tamoxifen-inducible transgenic mice. In this setting, Tbx3 efficiently suppressed the working myocardial gene program including suppression of the fast conducting gap junction genes (Gja1, Gja5), the cardiac sodium channel gene (Scn5a), and the inward rectifying potassium channel genes (Kcnj2, Kcnj3, Kcnj12). However, Tbx3 overexpression failed to induce ectopic pacemaker activity in isolated left atria. Lentiviral Tbx3 overexpression in neonatal rat ventricular myocytes further confirmed these outcomes [55]. In search of alternative factors, Kapoor et al., screened several important SAN transcription factors (Shox2, Tbx3, Tbx5, Tbx18, and Tbx20) and assessed their ability to increase spontaneous beating rates in neonatal rat ventricular CMs. This assay identified Tbx18 as a promising candidate. Follow-up studies in guinea pigs that received Tbx18 via adenoviral delivery into the left ventricular apex, indeed confirmed ectopic pacemaker function at the injection site. Importantly the newly formed iSAN cells resembled native SAN cells in their molecular signature, morphology, and function [56]. Motivated by these results, further validation was performed in a porcine model of complete AV-block. Here, Tbx18 was overexpressed in the AVB region, which generated effective biological pacemaker function for about a week. Thereafter biological pacemaker function was slowly lost. This could in part relate to the temporal expression kinetics of adenoviral gene transfer, yet the prior guinea pig study indicated that some degree of pacemaker function persists after loss of Tbx18 expression, probably resulting from partial maintenance of the reprogrammed state [57]. Additional in vitro studies indicated that epithelial-to-mesenchymal transition occurred in Tbx18 overexpressing cells, possibly contributing to a loss of reprogrammed cells from the injection. Although this imposed a new challenge, preliminary studies indicate that it may also provide an important avenue for further optimization as ongoing studies indicate that inhibition of EMT can be used to stabilize Tbx18-induceed pacemaker activity [58,59]. Although such an approach still requires further validation in long-term large animal studies, it supports the promising direction of TF-based biological pacemakers.

The above-mentioned studies provide us with experimental evidence on how forced expression of specific TFs in CMs drives them towards pacemaker-like phenotypes. This knowledge may also be applied to directly reprogram fibroblasts towards pacemaker-like cells. A first attempt in this direction has been reported by Nam and colleagues. To optimize their approach, they used the Hcn4-GFP reporter mouse line (with high GFP expression in the SAN) to screen a pool of 20 TFs [9]. With this group of TFs, they aimed to induce GFP expression in Hcn4-GFP transgenic fibroblasts. A four-factor (Gata6, Tbx3, Tbx5 and Rarg or Rxra) combination induced maximum GFP expression but failed to generate excitable cells, illustrating that they did not succeed in generating functional pacemaker cells. They also tested the GMTH cocktail, which produced a mixed population of immature CMs having atrial, ventricular and pacemaker phenotypes (approximately 33% each). This outcome suggests that the induction of a pacemaker phenotype is clearly feasible although significant optimization is still needed [51].

In addition to efforts towards the generation of pacemaker cells, attempts have been made to induce other pacemaker and CCS phenotypes by direct reprogramming. Notch signaling has been implied to be crucial in CCS development. Rentschler and colleagues have used a loss-of-function and gain-of-function approach to demonstrate the crucial role of Notch signaling in AV canal (AVC) formation during CCS formation in mouse [60]. Mlc2vCre/+DN-MAML (dominant-negative truncated form of the Notch co-activating mastermind-like protein) mice were used in which DN-MAML inhibits Notch receptors leading to loss of Notch signaling in AVC and ventricular myocardium. This resulted in AV node size reduction, specific loss of Cx30.2 expressing slow conducting cells and impaired maturation of AVC myocardium. In Mlc2v^Cre/+^; NICD (Notch intracellular domain) transgenic mice, Notch-activated hearts exhibited an enlarged AVN region. Histological and electrophysiological examination revealed the presence of additional accessory conduction pathways between the atria and ventricles leading to ventricular pre-excitation [60]. In a subsequent study, the same group demonstrated that ectopic Notch activation (using Mlc2v^Cre/+^; NICD) drives CM transdifferentiation towards a CCS phenotype [61]. Gene expression profiling of adult Notch-activated hearts indicated upregulation of several conduction chamber-specific genes (Cntn2, Cx30.2, Nkx2-5, Hcn1, Scn5a, Tbx5) when compared to control hearts. Electrophysiological properties of the individual isolated Notch-activated ventricular myocytes, to some degree resembled those of Purkinje cells. To further investigate the effect of transient Notch activation in vitro, perinatal mouse ventricular CMs were exposed to adenoviral overexpression of the Notch intracellular domain. This resulted in transcriptional upregulation of Notch targets (Hrt1, Hrt2, Hrt3, and Jag1), and the conduction-specific genes Cx40, Cntn2 and Hcn2 [61]. These two studies underline the ability of Notch signaling in the regulation of cardiac conduction cell fate decisions. Canonical Wnt signaling also regulates AVC formation during embryonic heart development. Disruption of canonical Wnt signaling resulted in loss of AVC myocardium and ectopic Wnt signaling activation resulted in the formation of AV-junction like tissue [62]. Moreover, ectopic expression of the paracrine factor Neuregulin-1, has been shown to convert day 8.5 to 10.5 embryonic CMs to CCS-like cells. This transdifferentiation response of CMs to Neurugulin-1 seemed to decline with more advanced embryonic development, as was shown using a CCS-specific lacZ reporter mice [63]. Moreover, a recent study identified Etv1, a neuregulin-1 responsive gene as a crucial factor in gene regulation of the CCS. Etv1 knock out mice showed reduced Scn5a, Nkx2-5 and Cx40 expression leading to developmental abnormalities and CCS defects [64]. Taken together, these studies suggest how to use our emerging understanding of molecular pathways during heart development to design direct reprogramming strategies supporting transdifferentiation towards pacemaker and CCS phenotypes.

## 4. Vector Systems Employed in Direct Reprogramming

Several viral vector systems have been used for the delivery of lineage-specific factors to support direct reprogramming in vitro and in vivo. When these vectors are considered for repair of the pacemaker and the CCS, they should ideally (i) target specific components of the CCS, (ii) have absent-to-minimal pathogenicity, (iii) be none or minimal immunogenic, (iv) harbor limited species-specificity (i.e., usable in different animal models and human cells), and (v) have a high transduction efficiency. There have been significant advancements in developing viral vectors that can efficiently deliver gene of interest into target cells. Adenoviral vectors have been a popular delivery method for temporary gene expression in the heart and have successfully been used to reprogram embryonic fibroblasts towards pluripotent stem cells [65]. Adeno Associated Virus (AAV) vectors have been widely used in cardiac gene therapy studies aiming for long-term transgene expression, but use for the overexpression of reprogramming factors has been limited due to the suboptimal transduction of fibroblasts. To improve fibroblast transduction, various mutations have successfully been introduced into the AAV2 capsid [66]. To some extent, retroviral vectors have been ideal for applications in myocardial regeneration in settings of MI injury, taking advantages of their selective transduction of rapidly dividing cells, thereby preferably targeting fibroblasts in the setting of acute MI. A potential downside of the retroviral vector system is its transgene integration, which could impose risks related to disruption of endogenous gene expression and/or insertional mutagenesis. Moreover, both in vitro and in vivo reprogramming efficiencies have been relatively low. To address this, a recent study investigated use of the non-integrating Sendai virus to carry GMT-based reprogramming. Using this method, the investigators achieved a 100-fold higher reprogramming as compared to the standard retroviral approach. This increase in reprogramming efficiency has been attributed to the higher transduction efficiency of the Sendai virus vector. An additional advantage of the Sendai virus system is that it appeared to provide effective reprogramming in both mouse and human CFs, making this vector potentially more useful to switch across different species (including large animals) while working towards applications in humans [49].

## 5. Future Perspectives

**Figure 1:**
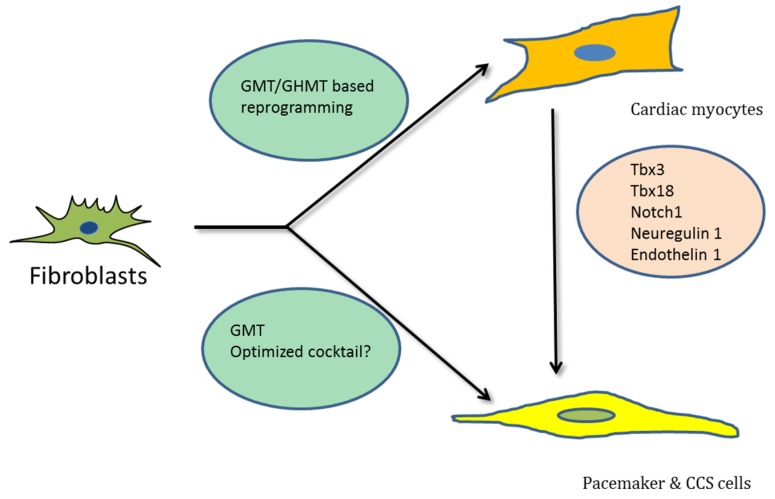
Schematic representation showing transdifferentiation of fibroblasts into different cardiac cell types by various transcription factors, respectively.

Direct reprogramming holds substantial promise for the regeneration of myocardium and restoration of cardiac pacemaker and CCS function (Figure 1). To achieve this goal, it will be crucial to generate sub-type-specific cells that can recapitulate all the structural, functional and electro-physiological aspects of working myocardium, pacemaker and CCS cells. Previous studies indicated that transdifferentiation towards iCMs generates at least three distinct immature sub-populations (i.e., iAMs, iVMs, iPMs [51]). Yet this may be a rather arbitrary classification of a broad spectrum of different subtypes, also including fibroblasts, intermediate fibroblasts, pre-iCMs and iCMs [40]. This observation further emphasizes the need for further improvements in existing direct reprogramming methods that can lead to the generation of the desired sub-types with higher efficiency and-specificity.

The embryonic development of SAN and CCS clearly involves highly regulated and precisely controlled stage-specific TF expression, signaling molecules and molecular pathways [52,60,61]. This illustrates the importance of expression and timing of factors required for proper development of the CCS. Similar approaches could be adopted within the various reprogramming approaches towards CCS sub-types. Most of the direct cardiac reprogramming studies thus far have employed constitutive overexpression of core cardiac TFs in fibroblasts. A vector system that allows temporal overexpression of TFs could potentially be used to improve outcomes. In this respect, the doxycycline (Dox)-inducible vector system is among the most widely used systems providing for temporal control of transgene expression. Ieda et al., used a poly-cistronic Dox-inducible cardiac reprogramming vector to reprogram mouse fibroblasts to iCMs in vitro. Using Dox-inducible-Hand2 along with constitutive expression of GMT revealed that Hand2 is crucial from the first day of reprogramming. Hand2 is shown to suppress cell-cycle promoting genes and also physically interacts with GMT to activate cardiac gene expression [67]. However, these inducible vectors are not free from disadvantages. The use of Dox itself has been shown to affect endogenous gene expression, which can compromise outcomes [68]. In addition, the toxicity of inducer molecules itself requires further optimization and validation. 

A relatively un-explored aspect in direct reprogramming is the translation to large animal testing. Given the reasonable efficacy of Sendai-GMT-based reprogramming, it would be useful to learn more about how effective such a strategy is in a clinically relevant large animal model. With regard to large animal testing of direct reprogramming approaches aimed at regeneration of the pacemaker and the CCS, the recent Tbx18 study [56] has provided an intriguing example. It showed that clinically available catheter-based delivery methods are ready to deliver reprogramming vectors to the proximal ventricular CCS. Moreover, comparable catheter delivery methods have been employed to deliver a variety of experimental gene therapies to the atrial, AVN, bundle of His and left bundle branch [69,70,71,72]. These methods have important similarities with today’s electrophysiological procedures and will therefore be easily integrated into clinical practice.

There are several potential target patient populations being considered for initial clinical studies. An obvious target cohort includes patients with acute MI as this has been the setting where direct reprogramming has been most extensively studied. With regard to dysfunction of the pacemaker and the CCS, demand pacing of the ventricle is expected to become feasible with currently available technology. Similar to other gene therapy-based approaches to biological pacing [71,73,74], this can be used as an adjuvant to, or eventual replacement of electronic pacing, introducing unique features such as improved autonomic responsiveness and optimal cardiac output.

## 6. Conclusions

Currently available experimental data shows significant promise for the general concept of direct in vivo reprogramming of fibroblasts. However, current methods need further refinement aiming for higher reprogramming efficiencies and more pure phenotypes to better recapitulate cardiac function in settings of myocardial repair and regeneration of the pacemaker and CCS.

## Figures and Tables

**Table 1:** Comparison of functional outcomes of in vivo direct reprogramming studies aimed to regenerate the mouse myocardium.

Reprogramming Factors	Weeks Post MI	Ejection Fraction	Stroke Volume	Scar Area (%)	References
G/M/T vs. dsRed group	12 w	~32% vs. 22%	~42 mL/min vs. 30 mL/min	~18% vs. 40%	[19]
G/M/T/H vs. GFP group	12 w	~58% vs. 30%	~55 µL vs. 40 µL	~18% vs. 40%	[21]
MGT vs. G/M/T	8 w	~38% vs. 24%	ND	~18% vs. 28% *	[20]

* Time point 4 weeks post Myocardial infarction. G: Gata4, M: Mef2C, T: Tbx5, H: Hand2, MGT: Single poly-cistronic vector encoding Mef2c, Gata4, Tbx5. ND: not defined.

**Table 2:** Comparison of Human iCM generation in vitro.

Reprogramming Factors	Cell Type	Reprogramming Read-Out	Reprogramming Efficiency	Beating Cells	References
Gata4, Hand2, Tbx5, Myocardin, miR-1, miR-133	Human neonatal FFs, adult CFs and DFs	cTNT	13%	+ (Rare)	[45]
GMT, Mesp1, Myocardin	Human CFs and DFs	Multiple cardiac gene expression, sarcomeric organization structure and calcium oscillations	6%	-	[46]
GMT-Mesp1 or GMT, miR-133a	Mouse & Human CFs	a-actinin protein, c-TnT calcium oscillations	10% (mouse) & 8% (human)	+	[47]
GMT, Hand2, Myocardin or GMT, miR-590	Porcine & Human CFs	cTNT	5%	+	[48]
SeV-GMT/H	Human CFs	cTNT	15%	+	[49]
GMT, ESRRG, MESP1, Myocardin, ZFPM2	Human ESC derived fibroblasts, neonatal and skin fibroblasts	cTNT	5%	-	[50]
GMT, ESRRG, MESP1, Myocardin, ZFPM2, TGFβ inhibitor, Wnt inhibitor	Adult HCF cell line	cTNT	12%	+	[51]

FFs: Foreskin fibroblasts; CFs: Cardiac Fibroblasts; DFs: Dermal Fibroblasts; SeV: Sendai virus; ESC embryonic stem cells.

## Abbreviations

TFs	Transcription factors
CMs	Cardiac myocytes
iCMs	induced cardiac myocytes
Micro RNAs	miRNAs
MCFs	Mouse cardiac fibroblasts
MEFs	Mouse embryonic fibroblasts
TTFs	Tail tip fibroblasts
GMT	Gata4, Mef2c, Tbx5
iAMs	induced atrial myocytes
iVMs	induced ventricular myocytes
iPMs	induced pacemaker myocytes
HCFs	Human cardiac fibroblasts
CCS	cardiac conduction system

## References

[1] Takahashi K., Yamanaka S. (2006). Induction of pluripotent stem cells from mouse embryonic and adult fibroblast cultures by defined factors. Cell.

[2] Devalla H.D., Schwach V., Ford J.W., Milnes J.T., El-Haou S., Jackson C., Gkatzis K., Elliott D.A., Chuva de Sousa Lopes S.M., Mummery C.L. (2015). Atrial-like cardiomyocytes from human pluripotent stem cells are a robust preclinical model for assessing atrial-selective pharmacology. EMBO Mol. Med..

[3] Karakikes I., Senyei G.D., Hansen J., Kong C.W., Azeloglu E.U., Stillitano F., Lieu D.K., Wang J., Ren L., Hulot J.S. (2014). Small molecule-mediated directed differentiation of human embryonic stem cells toward ventricular cardiomyocytes. Stem Cells Transl. Med..

[4] Protze S.I., Liu J., Nussinovitch U., Ohana L., Backx P.H., Gepstein L., Keller G.M. (2017). Sinoatrial node cardiomyocytes derived from human pluripotent cells function as a biological pacemaker. Nat. Biotechnol..

[5] Yamakawa H., Ieda M. (2015). Strategies for heart regeneration: Approaches ranging from induced pluripotent stem cells to direct cardiac reprogramming. Int. Heart J..

[6] Zhou Q., Brown J., Kanarek A., Rajagopal J., Melton D.A. (2008). In vivo reprogramming of adult pancreatic exocrine cells to beta-cells. Nature.

[7] Sekiya S., Suzuki A. (2011). Direct conversion of mouse fibroblasts to hepatocyte-like cells by defined factors. Nature.

[8] Vierbuchen T., Ostermeier A., Pang Z.P., Kokubu Y., Sudhof T.C., Wernig M. (2010). Direct conversion of fibroblasts to functional neurons by defined factors. Nature.

[9] Ieda M., Fu J.D., Delgado-Olguin P., Vedantham V., Hayashi Y., Bruneau B.G., Srivastava D. (2010). Direct reprogramming of fibroblasts into functional cardiomyocytes by defined factors. Cell.

[10] Boyett M.R., Inada S., Yoo S., Li J., Liu J., Tellez J., Greener I.D., Honjo H., Billeter R., Lei M. (2006). Connexins in the sinoatrial and atrioventricular nodes. Adv. Cardiol..

[11] Efimov I.R., Fedorov V.V., Joung B., Lin S.F. (2010). Mapping cardiac pacemaker circuits: methodological puzzles of the sinoatrial node optical mapping. Circ. Res..

[12] Yaniv Y., Lakatta E.G., Maltsev V.A. (2015). From two competing oscillators to one coupled-clock pacemaker cell system. Front. Physiol..

[13] Sneddon J.F., Camm A.J. (1992). Sinus node disease. Current concepts in diagnosis and therapy. Drugs.

[14] Vardas P.E., Auricchio A., Blanc J.J., Daubert J.C., Drexler H., Ector H., Gasparini M., Linde C., Morgado F.B., Oto A. (2007). Guidelines for cardiac pacing and cardiac resynchronization therapy. The Task Force for Cardiac Pacing and Cardiac Resynchronization Therapy of the European Society of Cardiology. Developed in collaboration with the European Heart Rhythm Association. Europace.

[15] Tellez J.O., McZewski M., Yanni J., Sutyagin P., Mackiewicz U., Atkinson A., Inada S., Beresewicz A., Billeter R., Dobrzynski H. (2011). Ageing-dependent remodelling of ion channel and Ca^2+^ clock genes underlying sino-atrial node pacemaking. Exp. Physiol..

[16] Csepe T.A., Kalyanasundaram A., Hansen B.J., Zhao J., Fedorov V.V. (2015). Fibrosis: A structural modulator of sinoatrial node physiology and dysfunction. Front. Physiol..

[17] Boink G.J., Christoffels V.M., Robinson R.B., Tan H.L. (2015). The past, present, and future of pacemaker therapies. Trends Cardiovasc. Med..

[18] Takeuchi J.K., Bruneau B.G. (2009). Directed transdifferentiation of mouse mesoderm to heart tissue by defined factors. Nature.

[19] Qian L., Huang Y., Spencer C.I., Foley A., Vedantham V., Liu L., Conway S.J., Fu J.D., Srivastava D. (2012). In vivo reprogramming of murine cardiac fibroblasts into induced cardiomyocytes. Nature.

[20] Ma H., Wang L., Yin C., Liu J., Qian L. (2015). In vivo cardiac reprogramming using an optimal single polycistronic construct. Cardiovasc. Res..

[21] Song K., Nam Y.J., Luo X., Qi X., Tan W., Huang G.N., Acharya A., Smith C.L., Tallquist M.D., Neilson E.G. (2012). Heart repair by reprogramming non-myocytes with cardiac transcription factors1. Nature.

[22] Chen J.X., Krane M., Deutsch M.A., Wang L., Rav-Acha M., Gregoire S., Engels M.C., Rajarajan K., Karra R., Abel E.D. (2012). Inefficient reprogramming of fibroblasts into cardiomyocytes using Gata4, Mef2c, and Tbx5. Circ. Res..

[23] Protze S., Khattak S., Poulet C., Lindemann D., Tanaka E.M., Ravens U. (2012). A new approach to transcription factor screening for reprogramming of fibroblasts to cardiomyocyte-like cells. J. Mol. Cell. Cardiol..

[24] Addis R.C., Ifkovits J.L., Pinto F., Kellam L.D., Esteso P., Rentschler S., Christoforou N., Epstein J.A., Gearhart J.D. (2013). Optimization of direct fibroblast reprogramming to cardiomyocytes using calcium activity as a functional measure of success. J. Mol. Cell. Cardiol..

[25] Zhou H., Dickson M.E., Kim M.S., Bassel-Duby R., Olson E.N. (2015). Akt1/protein kinase B enhances transcriptional reprogramming of fibroblasts to functional cardiomyocytes. Proc. Natl. Acad. Sci. USA.

[26] Zhou H., Morales M.G., Hashimoto H., Dickson M.E., Song K., Ye W., Kim M.S., Niederstrasser H., Wang Z., Chen B. (2017). ZNF281 enhances cardiac reprogramming by modulating cardiac and inflammatory gene expression. Genes Dev..

[27] Lin T., Ambasudhan R., Yuan X., Li W., Hilcove S., Abujarour R., Lin X., Hahm H.S., Hao E., Hayek A. (2009). A chemical platform for improved induction of human iPSCs. Nat. Methods.

[28] Maherali N., Hochedlinger K. (2009). Tgfbeta signal inhibition cooperates in the induction of iPSCs and replaces Sox2 and cMyc. Current Biol..

[29] Lian X., Hsiao C., Wilson G., Zhu K., Hazeltine L.B., Azarin S.M., Raval K.K., Zhang J., Kamp T.J., Palecek S.P. (2012). Robust cardiomyocyte differentiation from human pluripotent stem cells via temporal modulation of canonical Wnt signaling. Proc. Natl. Acad. Sci. USA.

[30] Ifkovits J.L., Addis R.C., Epstein J.A., Gearhart J.D. (2014). Inhibition of TGFbeta signaling increases direct conversion of fibroblasts to induced cardiomyocytes. PLoS ONE.

[31] Zhao Y., Londono P., Cao Y., Sharpe E.J., Proenza C., O’Rourke R., Jones K.L., Jeong M.Y., Walker L.A., Buttrick P.M. (2015). High-efficiency reprogramming of fibroblasts into cardiomyocytes requires suppression of pro-fibrotic signalling. Nat. Commun..

[32] Mohamed T.M., Stone N.R., Berry E.C., Radzinsky E., Huang Y., Pratt K., Ang Y.S., Yu P., Wang H., Tang S. (2017). Chemical Enhancement of In Vitro and In Vivo Direct Cardiac Reprogramming. Circulation.

[33] Liu Z., Chen O., Zheng M., Wang L., Zhou Y., Yin C., Liu J., Qian L. (2016). Re-patterning of H3K27me3, H3K4me3 and DNA methylation during fibroblast conversion into induced cardiomyocytes. Stem Cell Res..

[34] Jacobs J.J., Kieboom K., Marino S., DePinho R.A., van Lohuizen M. (1999). The oncogene and Polycomb-group gene bmi-1 regulates cell proliferation and senescence through the ink4a locus. Nature.

[35] Park I.K., Morrison S.J., Clarke M.F. (2004). Bmi1, stem cells, and senescence regulation. J. Clin. Investig..

[36] Zhou Y., Wang L., Vaseghi H.R., Liu Z., Lu R., Alimohamadi S., Yin C., Fu J.D., Wang G.G., Liu J. (2016). Bmi1 Is a Key Epigenetic Barrier to Direct Cardiac Reprogramming. Cell Stem Cell.

[37] Wamstad J.A., Alexander J.M., Truty R.M., Shrikumar A., Li F., Eilertson K.E., Ding H., Wylie J.N., Pico A.R., Capra J.A. (2012). Dynamic and coordinated epigenetic regulation of developmental transitions in the cardiac lineage. Cell.

[38] Liu L., Lei I., Karatas H., Li Y., Wang L., Gnatovskiy L., Dou Y., Wang S., Qian L., Wang Z. (2016). Targeting Mll1 H3K4 methyltransferase activity to guide cardiac lineage specific reprogramming of fibroblasts. Cell Discov..

[39] Wang L., Liu Z., Yin C., Zhou Y., Liu J., Qian L. (2015). Improved Generation of Induced Cardiomyocytes Using a Polycistronic Construct Expressing Optimal Ratio of Gata4, Mef2c and Tbx5. J. Vis. Exp..

[40] Liu Z., Wang L., Welch J.D., Ma H., Zhou Y., Vaseghi H.R., Yu S., Wall J.B., Alimohamadi S., Zheng M. (2017). Single-cell transcriptomics reconstructs fate conversion from fibroblast to cardiomyocyte. Nature.

[41] Zhou Y., Alimohamadi S., Wang L., Liu Z., Wall J.B., Yin C., Liu J., Qian L. (2018). A Loss of Function Screen of Epigenetic Modifiers and Splicing Factors during Early Stage of Cardiac Reprogramming. Stem Cells Int..

[42] Purvis N., Bahn A., Katare R. (2015). The Role of MicroRNAs in Cardiac Stem Cells. Stem Cells Int..

[43] Gangaraju V.K., Lin H. (2009). MicroRNAs: Key regulators of stem cells. Nat. Rev. Mol. Cell. Biol..

[44] Jayawardena T.M., Egemnazarov B., Finch E.A., Zhang L., Payne J.A., Pandya K., Zhang Z., Rosenberg P., Mirotsou M., Dzau V.J. (2012). MicroRNA-mediated in vitro and in vivo direct reprogramming of cardiac fibroblasts to cardiomyocytes. Circ. Res..

[45] Nam Y.J., Song K., Luo X., Daniel E., Lambeth K., West K., Hill J.A., DiMaio J.M., Baker L.A., Bassel-Duby R. (2013). Reprogramming of human fibroblasts toward a cardiac fate. Proc. Natl. Acad. Sci. USA.

[46] Wada R., Muraoka N., Inagawa K., Yamakawa H., Miyamoto K., Sadahiro T., Umei T., Kaneda R., Suzuki T., Kamiya K. (2013). Induction of human cardiomyocyte-like cells from fibroblasts by defined factors. Proc. Natl. Acad. Sci. USA.

[47] Muraoka N., Yamakawa H., Miyamoto K., Sadahiro T., Umei T., Isomi M., Nakashima H., Akiyama M., Wada R., Inagawa K. (2014). MiR-133 promotes cardiac reprogramming by directly repressing Snai1 and silencing fibroblast signatures. EMBO J..

[48] Singh V.P., Mathison M., Patel V., Sanagasetti D., Gibson B.W., Yang J., Rosengart T.K. (2016). MiR-590 Promotes Transdifferentiation of Porcine and Human Fibroblasts Toward a Cardiomyocyte-Like Fate by Directly Repressing Specificity Protein 1. J. Am. Heart Assoc..

[49] Miyamoto K., Akiyama M., Tamura F., Isomi M., Yamakawa H., Sadahiro T., Muraoka N., Kojima H., Haginiwa S., Kurotsu S. (2018). Direct In Vivo Reprogramming with Sendai Virus Vectors Improves Cardiac Function after Myocardial Infarction. Cell Stem Cell.

[50] Fu J.D., Stone N.R., Liu L., Spencer C.I., Qian L., Hayashi Y., Delgado-Olguin P., Ding S., Bruneau B.G., Srivastava D. (2013). Direct reprogramming of human fibroblasts toward a cardiomyocyte-like state. Stem Cell Rep..

[51] Nam Y.J., Lubczyk C., Bhakta M., Zang T., Fernandez-Perez A., McAnally J., Bassel-Duby R., Olson E.N., Munshi N.V. (2014). Induction of diverse cardiac cell types by reprogramming fibroblasts with cardiac transcription factors. Development.

[52] Van Weerd J.H., Christoffels V.M. (2016). The formation and function of the cardiac conduction system. Development.

[53] Hoogaars W.M., Tessari A., Moorman A.F., de Boer P.A., Hagoort J., Soufan A.T., Campione M., Christoffels V.M. (2004). The transcriptional repressor Tbx3 delineates the developing central conduction system of the heart. Cardiovasc. Res..

[54] Hoogaars W.M., Engel A., Brons J.F., Verkerk A.O., de Lange F.J., Wong L.Y., Bakker M.L., Clout D.E., Wakker V., Barnett P. (2007). Tbx3 controls the sinoatrial node gene program and imposes pacemaker function on the atria. Genes Dev..

[55] Bakker M.L., Boink G.J., Boukens B.J., Verkerk A.O., van den Boogaard M., den Haan A.D., Hoogaars W.M., Buermans H.P., de Bakker J.M., Seppen J. (2012). T-box transcription factor TBX3 reprograms mature cardiac myocytes into pacemaker-like cells. Cardiovasc. Res..

[56] Kapoor N., Liang W., Marban E., Cho H.C. (2013). Direct conversion of quiescent cardiomyocytes to pacemaker cells by expression of Tbx18. Nat. Biotechnol..

[57] Hu Y.F., Dawkins J.F., Cho H.C., Marban E., Cingolani E. (2014). Biological pacemaker created by minimally invasive somatic reprogramming in pigs with complete heart block. Sci. Transl. Med..

[58] Li J., Gonzalez S., Lehman J., Cho H.C. (2016). Stable and synchronous pacing generated from the Tbx18-induced pacemaker cells by epithelial-to-mesenchymal transition inhibition. Circulation.

[59] Kim N.K., Li J., Wolfson D., Fernandez N., Gu J., Han P., Grijalva S., Cho H.C. (2017). Stable in vivo ventricular pacing created by Tbx18-induced pacemaker cells upon inhibition of epithelial-to-mesenchymal transformation in an ambulatory rat model of complete atrioventricular block. Circulation.

[60] Rentschler S., Harris B.S., Kuznekoff L., Jain R., Manderfield L., Lu M.M., Morley G.E., Patel V.V., Epstein J.A. (2011). Notch signaling regulates murine atrioventricular conduction and the formation of accessory pathways. J. Clin. Investig..

[61] Rentschler S., Yen A.H., Lu J., Petrenko N.B., Lu M.M., Manderfield L.J., Patel V.V., Fishman G.I., Epstein J.A. (2012). Myocardial Notch signaling reprograms cardiomyocytes to a conduction-like phenotype. Circulation.

[62] Gillers B.S., Chiplunkar A., Aly H., Valenta T., Basler K., Christoffels V.M., Efimov I.R., Boukens B.J., Rentschler S. (2015). Canonical wnt signaling regulates atrioventricular junction programming and electrophysiological properties. Circ. Res..

[63] Rentschler S., Zander J., Meyers K., France D., Levine R., Porter G., Rivkees S.A., Morley G.E., Fishman G.I. (2002). Neuregulin-1 promotes formation of the murine cardiac conduction system. Proc. Natl. Acad. Sci. USA.

[64] Shekhar A., Lin X., Liu F.Y., Zhang J., Mo H., Bastarache L., Denny J.C., Cox N.J., Delmar M., Roden D.M. (2016). Transcription factor ETV1 is essential for rapid conduction in the heart. J. Clin. Investig..

[65] Zhou W., Freed C.R. (2009). Adenoviral gene delivery can reprogram human fibroblasts to induced pluripotent stem cells. Stem Cells.

[66] Li M., Jayandharan G.R., Li B., Ling C., Ma W., Srivastava A., Zhong L. (2010). High-efficiency transduction of fibroblasts and mesenchymal stem cells by tyrosine-mutant AAV2 vectors for their potential use in cellular therapy. Hum. Gene Ther..

[67] Umei T.C., Yamakawa H., Muraoka N., Sadahiro T., Isomi M., Haginiwa S., Kojima H., Kurotsu S., Tamura F., Osakabe R. (2017). Single-Construct Polycistronic Doxycycline-Inducible Vectors Improve Direct Cardiac Reprogramming and Can Be Used to Identify the Critical Timing of Transgene Expression. Int. J. Mol. Sci..

[68] Merentie M., Rissanen R., Lottonen-Raikaslehto L., Huusko J., Gurzeler E., Turunen M.P., Holappa L., Makinen P., Yla-Herttuala S. (2018). Doxycycline modulates VEGF-A expression: Failure of doxycycline-inducible lentivirus shRNA vector to knockdown VEGF-A expression in transgenic mice. PLoS ONE.

[69] Tse H.F., Xue T., Lau C.P., Siu C.W., Wang K., Zhang Q.Y., Tomaselli G.F., Akar F.G., Li R.A. (2006). Bioartificial sinus node constructed via in vivo gene transfer of an engineered pacemaker HCN Channel reduces the dependence on electronic pacemaker in a sick-sinus syndrome model. Circulation.

[70] Donahue J.K., Heldman A.W., Fraser H., McDonald A.D., Miller J.M., Rade J.J., Eschenhagen T., Marban E. (2000). Focal modification of electrical conduction in the heart by viral gene transfer. Nat. Med..

[71] Cingolani E., Yee K., Shehata M., Chugh S.S., Marban E., Cho H.C. (2012). Biological pacemaker created by percutaneous gene delivery via venous catheters in a porcine model of complete heart block. Heart Rhythm.

[72] Bucchi A., Plotnikov A.N., Shlapakova I., Danilo P., Kryukova Y., Qu J., Lu Z., Liu H., Pan Z., Potapova I. (2006). Wild-type and mutant HCN channels in a tandem biological-electronic cardiac pacemaker. Circulation.

[73] Boink G.J., Nearing B.D., Shlapakova I.N., Duan L., Kryukova Y., Bobkov Y., Tan H.L., Cohen I.S., Danilo P., Robinson R.B. (2012). Ca(2+)-stimulated adenylyl cyclase AC1 generates efficient biological pacing as single gene therapy and in combination with HCN2. Circulation.

[74] Boink G.J., Duan L., Nearing B.D., Shlapakova I.N., Sosunov E.A., Anyukhovsky E.P., Bobkov E., Kryukova Y., Ozgen N., Danilo P. (2013). HCN2/SkM1 gene transfer into canine left bundle branch induces stable, autonomically responsive biological pacing at physiological heart rates. J. Am. Coll. Cardiol..

